# Insights into the substrate binding mechanism of SULT1A1 through molecular dynamics with excited normal modes simulations

**DOI:** 10.1038/s41598-021-92480-w

**Published:** 2021-06-23

**Authors:** Balint Dudas, Daniel Toth, David Perahia, Arnaud B. Nicot, Erika Balog, Maria A. Miteva

**Affiliations:** 1 Inserm U1268 MCTR, CiTCoM UMR 8038 CNRS - University of Paris, Pharmacy Faculty of Paris, Paris, France; 2 Laboratoire de Biologie et Pharmacologie Appliquée, Ecole Normale Supérieure Paris-Saclay, UMR 8113, CNRS, Gif-sur-Yvette, France; 3 Department of Biophysics and Radiation Biology, Semmelweis University, Budapest, Hungary; 4 Inserm, Université de Nantes, Centre de Recherche en Transplantation et Immunologie, UMR 1064, ITUN, 44000 Nantes, France

**Keywords:** Chemical biology, Cheminformatics, Computational biology and bioinformatics, Protein function predictions, Protein structure predictions, Virtual drug screening

## Abstract

Sulfotransferases (SULTs) are phase II drug-metabolizing enzymes catalyzing the sulfoconjugation from the co-factor 3′-phosphoadenosine 5′-phosphosulfate (PAPS) to a substrate. It has been previously suggested that a considerable shift of SULT structure caused by PAPS binding could control the capability of SULT to bind large substrates. We employed molecular dynamics (MD) simulations and the recently developed approach of MD with excited normal modes (MDeNM) to elucidate molecular mechanisms guiding the recognition of diverse substrates and inhibitors by SULT1A1. MDeNM allowed exploring an extended conformational space of PAPS-bound SULT1A1, which has not been achieved up to now by using classical MD. The generated ensembles combined with docking of 132 SULT1A1 ligands shed new light on substrate and inhibitor binding mechanisms. Unexpectedly, our simulations and analyses on binding of the substrates estradiol and fulvestrant demonstrated that large conformational changes of the PAPS-bound SULT1A1 could occur independently of the co-factor movements that could be sufficient to accommodate large substrates as fulvestrant. Such structural displacements detected by the MDeNM simulations in the presence of the co-factor suggest that a wider range of drugs could be recognized by PAPS-bound SULT1A1 and highlight the utility of including MDeNM in protein–ligand interactions studies where major rearrangements are expected.

## Introduction

Drug metabolizing enzymes (DMEs) play a key role in the metabolism of endogenous molecules and the detoxification of xenobiotics and drugs^1–3^. Phase I metabolism includes hydrolysis, reduction, and oxidation reactions, while Phase II comprises mainly glucuronidation, sulfation, methylation, and glutathione conjugation reactions^4^. Sulfotransferases (SULTs) and UDP-glucuronosyltransferases are responsible for most of the Phase II reactions in the body, with the conjugation of approximately 40% of all drugs^5^. SULTs catalyze the sulfoconjugation from the co-factor 3′-phosphoadenosine 5′-phosphosulfate (PAPS) to a substrate hydroxyl or amino group^6–9^. DMEs are highly promiscuous, and the relations of their structural plasticity and substrate promiscuity have been widely studied^1,5,6,10–17^. SULTs show a broad substrate range, metabolizing a wide variety of endogenous compounds like steroids and polysaccharide chains, and participating in the bioactivation of a number of xenobiotics and drugs^7^.

**Figure 1:**
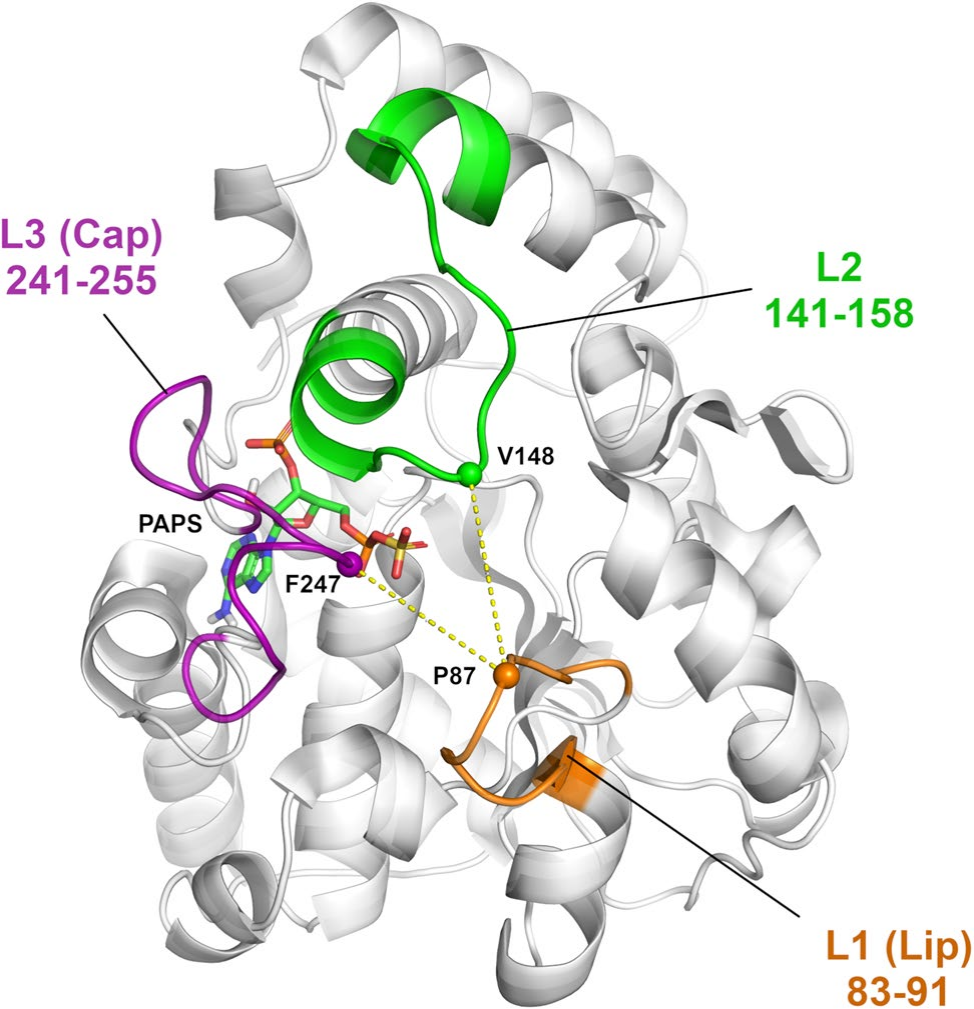
Crystal structure of SULT1A1*1, PDB ID: 4GRA^24^. PAP of 4GRA was replaced by PAPS which was retrieved from the structure of SULT1E1 (PDB ID: 1HY3^47^ containing PAPS) and inserted on the same position as that of the nucleotide in 4GRA; it is shown in sticks. The three loops covering the active site are indicated: L1 (“Lip”) in orange, L2 in green, and L3 (“Cap”) in magenta.

The molecular bases of substrate specificity, selectivity, and inhibition across different SULT isoforms, have been previously addressed^10,11,18–26^. These specificities have proven to be complex as relationships between SULTs pocket characteristics and substrate shape have shown not to be direct, since pocket shape and size have the potential to fluctuate upon substrate binding^22^. Structural displacements can alter the substrate-binding profiles, thus guide enzyme–substrate interactions. It has been demonstrated that the binding of PAPS causes a considerable shift in the PAPS binding domain of SULT, moving a strongly conserved 30-residue active site “Cap”, which covers both the nucleotide co-factor and the substrate-binding site, towards “closure” (Fig. 1). This large movement, called “gating”, was suggested to participate in an isomerization equilibrium rate controlling the potential of SULT to bind larger substrates^22,24,25,27^. However, sulfonation data for SULT2A1/raloxifene strikingly revealed that the enzyme was still capable of turnover^28^ with approximately 5% of SULT2A1 remaining in its open state even at saturating levels of PAPS^5,24^. These data demonstrate that the gating mechanism may not be dependent only on the co-factor binding and that the mechanism of substrate recognition and selectivity should be further elucidated.

Molecular dynamics (MD) simulations^29^ and more recent Normal Mode Analysis approaches^30,31^ have become major techniques in the arsenal of tools developed to investigate the mode of action of bioactive molecules. A recent approach called MDeNM (molecular dynamics with excited normal modes) has recently been developed using low-frequency normal mode directions in MD simulations^32^. This approach considers many different linear combinations of NM vectors, each used in an independent MD simulation in which the corresponding collective motion is kinetically excited. Therefore, a wide variety of large movements can be promoted straightforwardly, which would be costly by standard MD simulations. So far MDeNM has been used successfully to study large functional movements in several biological systems^33–36^.

In this study, we focused on SULT1A1^37^, which is the most abundant SULT in the human liver. The SULT1A1 enzyme is widely distributed throughout the body, with a high abundance in organs such as the liver, lung, platelets, kidney, and gastrointestinal tissues^38^. Human SULT1A1 exhibits a broad substrate range with specificity for small phenolic compounds, including the drugs acetaminophen and minoxidil, and pro-carcinogens such as N-hydroxy-aromatic and heterocyclicaryl amines^7^. To elucidate the gating mechanism guiding the recognition of diverse substrates, in this work, we employed the recently developed original approach of MDeNM^32^ to explore an extended conformational space of the PAPS-bound SULT1A1 (SULT1A1/PAPS), which has not been achieved up to now by using classical MD simulations^21–25^. The investigation of the generated ensembles combined with the docking of 132 SULT1A1 substrates and inhibitors shed new light on the substrate recognition and inhibitor binding mechanisms. The performed MD and MDeNM simulations of SULT1A1/PAPS as well as MD and docking simulations with the substrates estradiol and fulvestrant, previously suggested to undergo different binding mechanisms^24^, demonstrated that large conformational changes of the PAPS-bound SULT1A1 can occur. Such conformational changes could be sufficient to accommodate large substrates, e.g. fulvestrant, independently of the co-factor movements. Indeed, such structural displacements were successfully detected by the MDeNM simulations and suggest that a wider range of drugs could be recognized by PAPS-bound SULT1A1.

## Results and discussion

MDeNM simulations enable an extended sampling of the conformational space by running multiple short MD simulations during which motions described by a subset of low-frequency Normal Modes are kinetically excited^32^. Thus, MDeNM simulations of SULT1A1/PAPS would allow detecting “open”-like conformations of SULT1A1, previously generated by MD simulations performed in the absence of its bound co-factor PAP(S)^20,23–25^. PAPS was included in the co-factor binding site of SULT1A1 (see “Materials and methods” for details) and maintained bound to SULT1A1 in all our simulations, since it was demonstrated that the co-factor is required for the correct folding of the substrate-binding site. Previous crystal structures of co-factor-free SULT have shown significant unfolding of the key loop L3 (Fig. 1) covering the co-factor and substrate binding sites^11^. Here, the conformational sampling of SULT1A1/PAPS was performed by running: (1) three 200 ns long MD simulations with different initial velocity distributions and (2) the previously developed efficient simulation method, MDeNM^32^—with 240 replicas—that combines Normal Mode Analysis (NMA) and Molecular Dynamics. MDeNM performs several simultaneous MD simulations during which motions along different randomized linear combinations of the most relevant low-frequency normal modes are promoted in the form of a velocity increment. The starting crystallographic coordinates for SULT1A1*1 were taken from the Protein Data Bank^39^, PDB ID 4GRA^24^, containing the co-factor PAP. We replaced PAP with PAPS required for the sulfonation catalytic activity of SULT1A1. No substrates/inhibitors were included in the MD and MDeNM simulations to avoid possible ligand-induced biases of the SULT1A1/PAPS structure. The total simulation time was 600 ns for the MD and 48 ns for the MDeNM simulations (see the Methods for details).

### Structural analysis of the MD and MDeNM generated conformational ensembles

**Figure 2:**
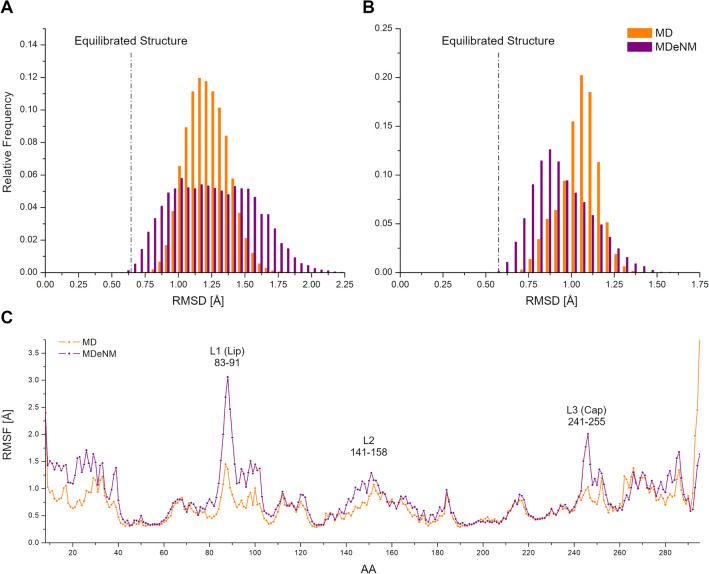
The Root Mean Square Deviation (RMSD) with respect to the crystal structure PDB ID: 4GRA of the MD (in orange) and MDeNM (in purple) generated structures of SULT1A in the presence of the PAPS. (**A**) Calculated on the binding pocket heavy atoms (the residues of the binding pocket are given in the Supporting Information and (**B**) on the backbone of the whole protein; (**C**) Root Mean Square Fluctuation (RMSF) of C_α_ atoms per amino acid residue (AA) in the MD (orange) and MDeNM (purple) conformational ensembles.

In order to identify similarities and differences in the conformational ensembles generated by the MD and MDeNM simulations, the Root Mean Square Deviation (RMSD) of the binding pocket (its residues are listed in the SI) was calculated with respect to the crystal structure (Fig. 2A). The MD conformations distribution covers an RMSD range between 0.75 Å and 1.75 Å with a clear peak around 1.2 Å with respect to the binding pocket of the starting crystal structure. The MDeNM conformations distribution of the binding pocket is more dispersed, even reaching conformations with a binding pocket deviating up to 2.25 Å from the crystal structure. Particularly, the region corresponding to RMSD values above 1.45 Å is more populated by MDeNM. The RMSD distribution of the whole protein backbone, calculated for the MDeNM conformations, showed a peak closer to the starting structure than that of the conformations generated by MD (Fig. 2B). However, the MDeNM simulations also generated conformations that deviate more from the crystal structure than those observed by MD, up to 1.5 Å. Larger deviations in the case of our MDeNM simulations originate from significant global movements of the protein. Larger deviations hence imply a more exhaustive conformational sampling, especially for the binding pocket. Our results suggest that MDeNM performed a more exhaustive conformational sampling of the SULT1A1 binding pocket while maintaining the protein’s overall structure closer to the starting structure.

The Root Mean Square Fluctuation (RMSF) of the Cα atoms was calculated to identify flexible protein regions of functional importance (Fig. 2C). Significant differences are visible at the gate (formed by loops L1, L2, and L3) of the binding pocket of SULT1A1 between conformational ensembles generated by the two methods. MDeNM particularly magnifies motions related to L1 (residues 83–91) and L3 (residues 241–255) and moderately related to L2 (residues 141–158). The fluctuation amplitude of the residues P87 and E246 at the tip of L1 and L3, respectively, is double in the case of MDeNM, indicating that MDeNM explores the gating motions to a greater extent. The Cap L3 has been suggested to play a key role in the gating mechanism of SULT1A1^24^ and SULT2A1^25,28^, fluctuating between a closed and an open isomer depending on the nucleotide-binding. L1 (also known as the “Lip”^40^) demonstrates a larger fluctuation than L3 by both MD and MDeNM, implying its involvement in the gating mechanism. Obviously, here the presence of PAPS stabilizes L3, which is known to be completely unfolded in the absence of bound co-factor^11^. Although the RMSF of both MD and MDeNM demonstrates the flexibility of L1, L2 and L3, larger movements of L1 and L3 are observed by the MDeNM simulations than by the MD.

**Figure 3:**
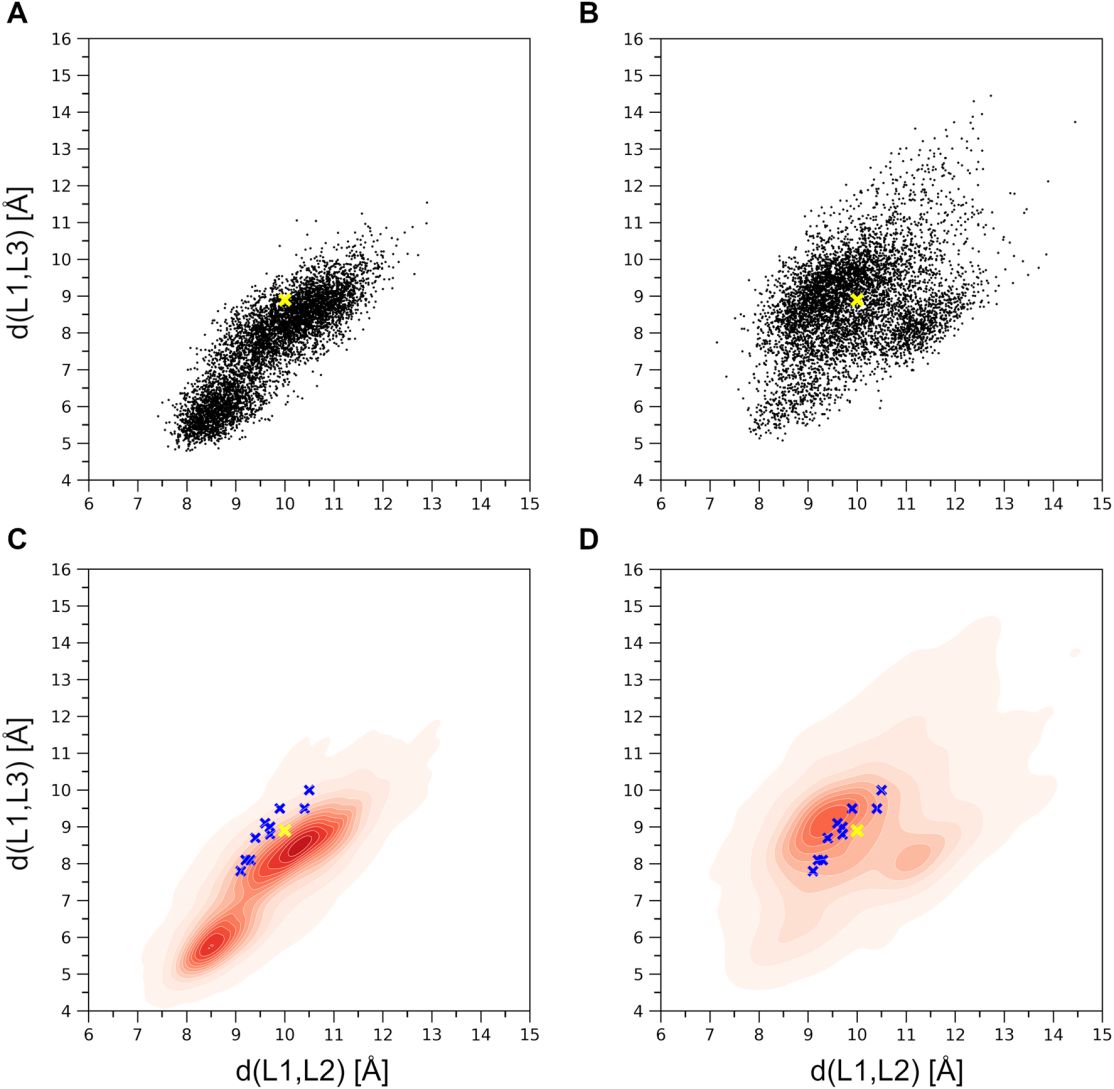
(**A**) Distribution of the d(L1,L2) and d(L1,L3) distances corresponding to the MD generated conformations; (**B**) Distribution corresponding to the MDeNM generated conformations; (**C**) Corresponding normalized distribution densities for the MD conformations and (**D**). for the MDeNM conformations. The available crystal structures (denoted by blue ‘x’-es) are plotted into the maps of (C, D); The location of the crystal structure (4GRA.pdb) is shown in yellow ‘x’.

The Cα atoms of residues P87, V148, and F247 representing each loop at their tip were selected to follow the relative motions and the gating mechanism of the three loops at the entrance to the binding pocket. Two distances, namely d(L1,L2) and d(L1,L3), were monitored corresponding to the distances d(P87Cα,V148Cα) and d(P87Cα,F247Cα) (see Fig. 1). The distribution of all generated conformations along these two distances can be seen in Fig. 3. Conformations reached by MD (Fig. 3A) exhibit a strong positive correlation (the correlation being 0.86) between d(L1,L2) and d(L1,L3), restricting thus the opening of the gate to occur along both distances at the same time. Interestingly, there are two dense regions in the MD conformations distribution, one lying close to the initial conformation (4GRA.pdb) denoted by yellow ‘x’, and another one corresponding to a more closed state. MD did not explore conformations having d(L1,L3) greater than 11.5 Å. The MDeNM distribution (Fig. 3B) is more widely spread and less restricted by the d(L1,L2) and d(L1,L3) correlation (the correlation being 0.40). MDeNM reaches conformations with the d(L1,L3) distance 3 Å beyond MD, up to 14.5 Å, corresponding to more widely open conformations, whereas MD maps densely populated tightly closed states. Both MD and MDeNM covered and reached far beyond the gate positions of L1, L2, and L3—both in the closing and the opening directions—of experimentally available conformations (the apo-forms of SULT1A1*1 and SULT1A1*2 without bound ligand PDB IDs 4GRA and 3U3J, respectively; the holo-forms of SULT1A1*2 with bound ligand PDB IDs: 1LS6, 2D06, 3U3M, 3U3O, 3U3R, 3U3K; and two ancestral variant b9 PDB IDs: 3QVU, 3QVV) (see Fig. 3C,D), which exhibit a very conserved overall structure with slight differences in their gate opening, the RMSD difference calculated on the Cα-s of the whole protein between any two experimental structures being less than 0.51 Å. The observed correlation between d(L1,L2) and d(L1,L3) in addition to the high RMSF values at L1, and visual inspection further confirmed the significant movements of L1 by the opening-closing of the gate, underlining the functional importance of L1 by SULT1A1 as proposed in the work of Rakers et al. for SULT 1E1^26^.

### Ensemble docking of SULT1A1 substrates and inhibitors

**Figure 4:**
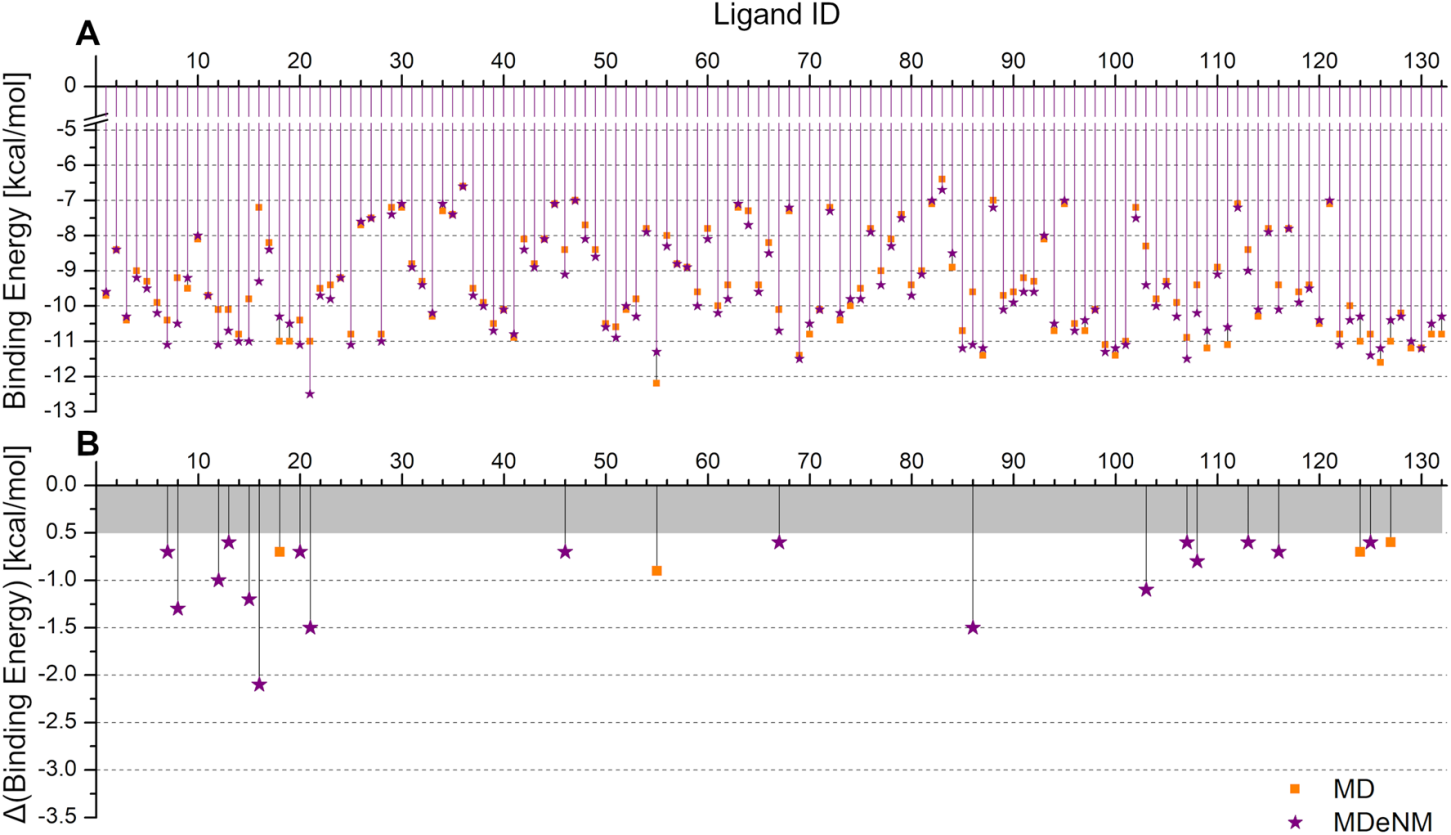
(**A**) The lowest binding energy (BE) per ligand resulting from the docking of the set of 132 known ligands to the ensemble of representative structures after clustering of SULT1A1/PAPS obtained from the MD (denoted by orange squares) and MDeNM (denoted by purple stars) simulations. (**B**) Differences between the best BEs retained for the MD and MDeNM conformations; for the better visualization, only differences larger than 0.5 kcal/mol are indicated.

The docking of 132 previously known substrates or inhibitors (collected in our previous work^10^ and^28,41^) was performed into the binding pocket of the conformations collected by MD and MDeNM to gain insight into the mechanism of SULT1A1-ligand interactions. First, both the MD and MDeNM generated conformations were clustered based on their binding pocket (see the list of residues in SI) to obtain a smaller, representative set of conformations to be used for the docking of all the ligands (see Methods for details). We performed docking on 94 MD and 86 MDeNM centroid SULT1A1/PAPS conformations. For each docking simulation, the best Binding Energy (BE) was retained. As different ligands can be accommodated in different binding pocket arrangements, for each ligand the best BE over the set of conformations have been taken; the results are plotted in Fig. 4A. Many ligands expressed similar docking behavior into the MD and MDeNM set of conformations, the average of the BEs over all the ligands being − 9.33 kcal/mol and − 9.49 kcal/mol, while the worst BE being − 6.4 kcal/mol and − 6.6 kcal/mol for MD and MDeNM, respectively. For some ligands, however, considerable differences were observed (see Fig. 4B). Most of these compounds (17 out of the 21 showing a difference greater than 0.5 kcal/mol) showed a more favorable BE when docked to the MDeNM set of conformations, demonstrating the benefit of including the MDeNM simulations in addition to MD. We compared the predicted and experimental binding energies reported in the literature for several SULT1A1 ligands (see in SI Table S1 and Fig. S1). Predicted binding energies (BE) were calculated by averaging the best scored Autodock Vina energies in the best 10 MD conformations and in the best 10 MDeNM conformations. The comparison between the experimental free binding energies and the scores calculated with Autodock Vina can be only qualitative, yet a correlation with a correlation coefficient R^2^ of 0.56 was obtained. Interestingly, the Vina scores distinguished between the low-affinity substrate p-nitrophenol with experimental BE of − 5.76 kcal/mol^42^ and the other higher affinity ligands.

To characterize the binding poses of the substrates, a criterion of having their acceptor hydroxyl or primary amino functional group in the vicinity of the sulfate group of the co-factor PAPS and the catalytic residue H108 was imposed. Docking positions and the corresponding BE of substrates with the d(O,S) or d(N,S) distance greater than 5 Å were rejected, and the best BE satisfying the distance criterion was taken (see in SI Fig. S2). For 22 out of the 26 compounds showing a difference greater than 0.5 kcal/mol with the applied distance criterion, docking into the MDeNM conformations outperformed the MD ones. The assessment of ligands for which there was a significant difference between MD and MDeNM (greater than 1 kcal/mol) revealed that most of the compounds for which MDeNM performed better were of big size, occupying a large volume in the binding pocket, and their poses corresponding to the best BE were accommodated within widely open SULT1A1/PAPS conformations. These conformations were either not generated or poorly populated by the MD simulations (see in SI Fig. S3).

### Implication of substrate binding and SULT1A1 flexibility for gating mechanism elucidation

**Figure 5:**
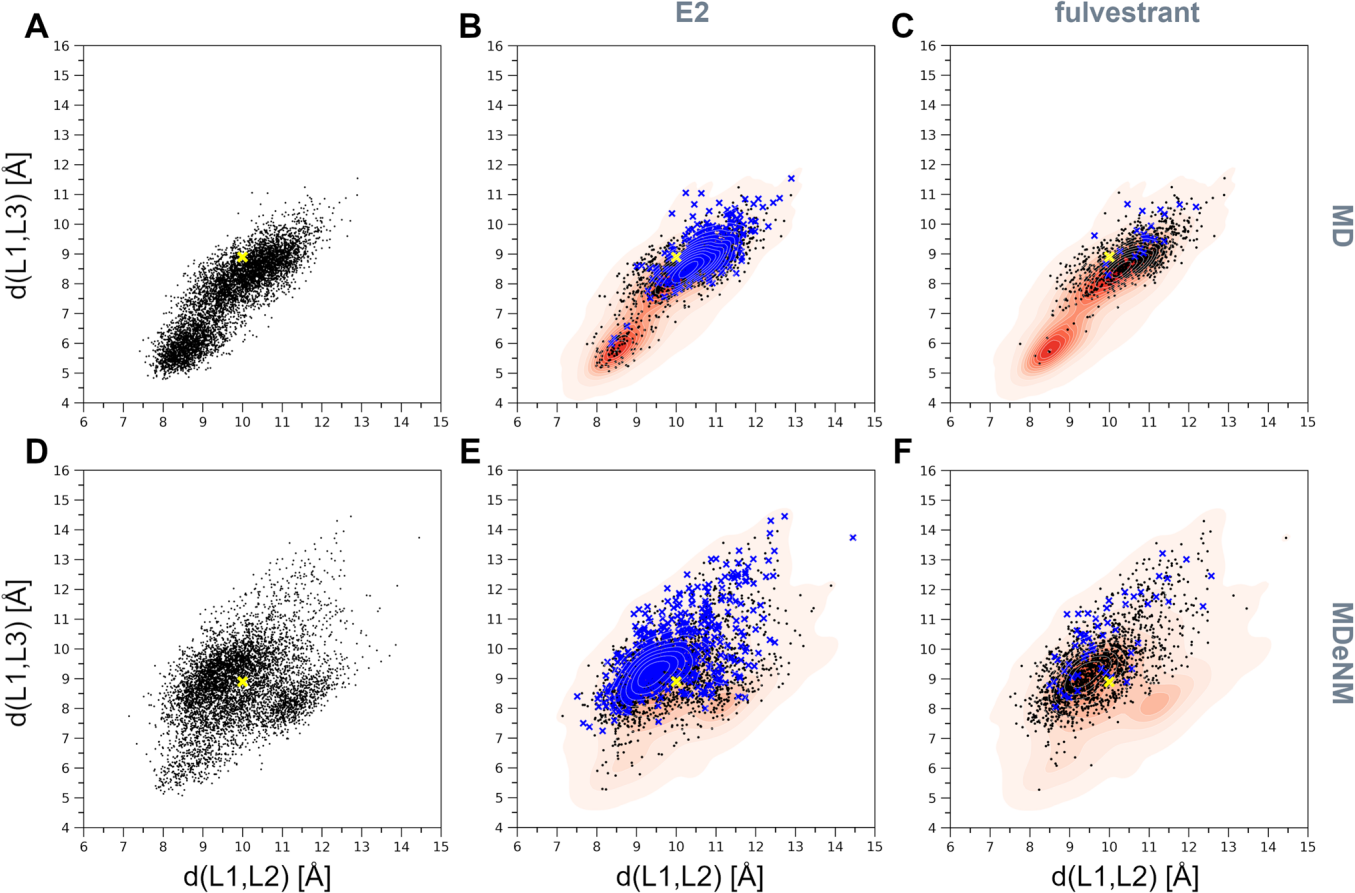
Distribution within the space defined by d(L1,L2) and d(L1,L3) distances for (**A**) the MD generated structures, (**B**) MD structures capable of accommodating competent E2 orientations, (**C**) MD structures capable of accommodating competent fulvestrant orientations; (**D**) the MDeNM generated structures, (**E**) MDeNM structures capable of accommodating competent E2 orientations, and (**F**) MDeNM structures capable of accommodating competent fulvestrant orientations. Conformations showing a BE stronger than -5 kcal/mol are indicated in black points and those showing a BE stronger than − 10 kcal/mol are indicated in blue ‘x’-es on parts B, C, E, and F. The initial crystal structure (4GRA.pdb) is shown in yellow ‘x’.

**Figure 6:**
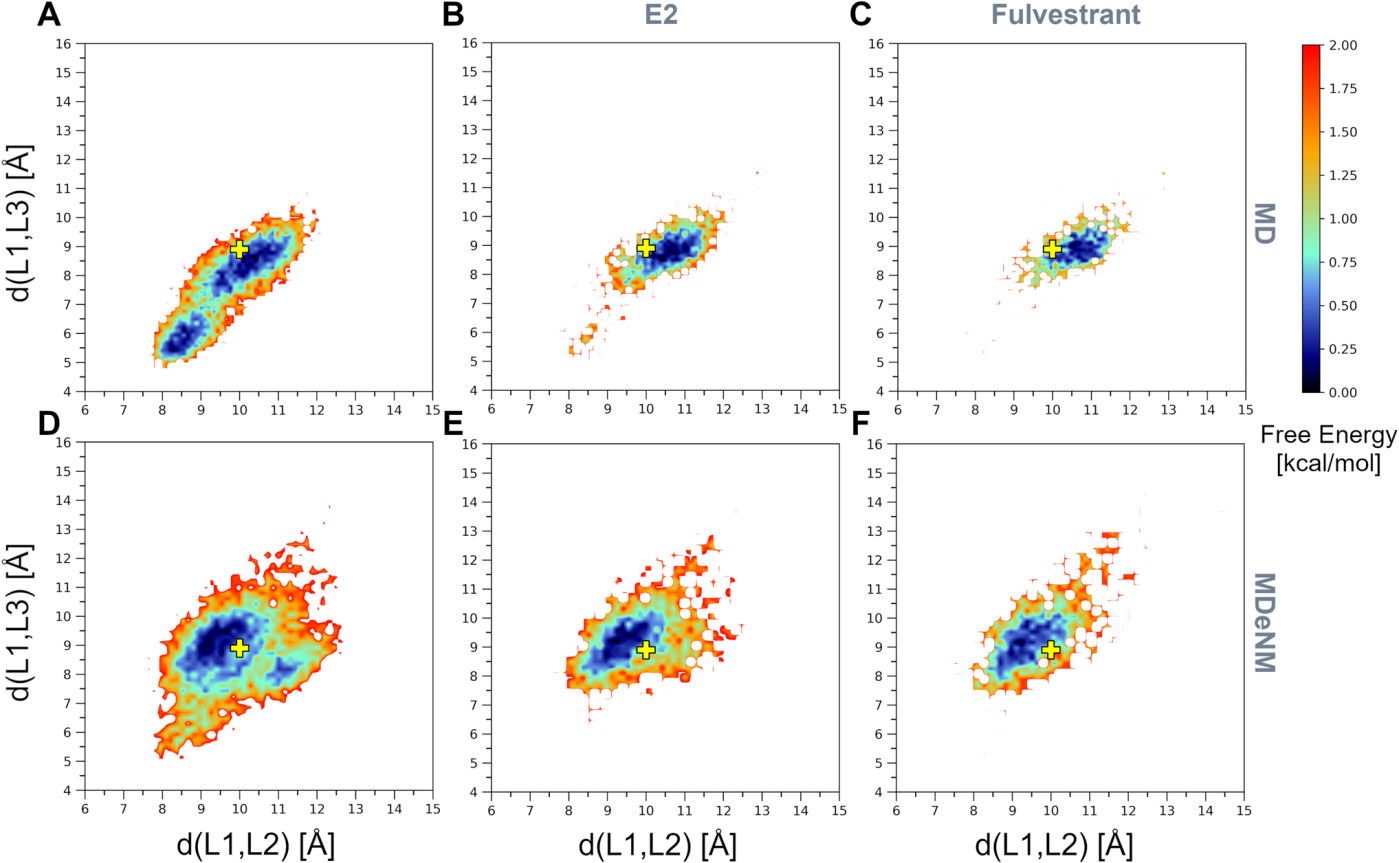
Free Energy Landscapes (FELs) in the space defined by the distances d(L1,L2) and d(L1,L3) of (**A**) the MD generated structures, (**B**) MD structures capable of accommodating competent E2 orientations, (**C**) MD structures capable of accommodating competent fulvestrant orientations; (**D**) the MDeNM generated structures, (**E**) MDeNM structures capable of accommodating competent E2 orientations, and (**F**) MDeNM structures capable of accommodating competent fulvestrant orientations. The initial crystal structure (4GRA.pdb) is denoted by yellow ‘ + ’.

**Figure 7:**
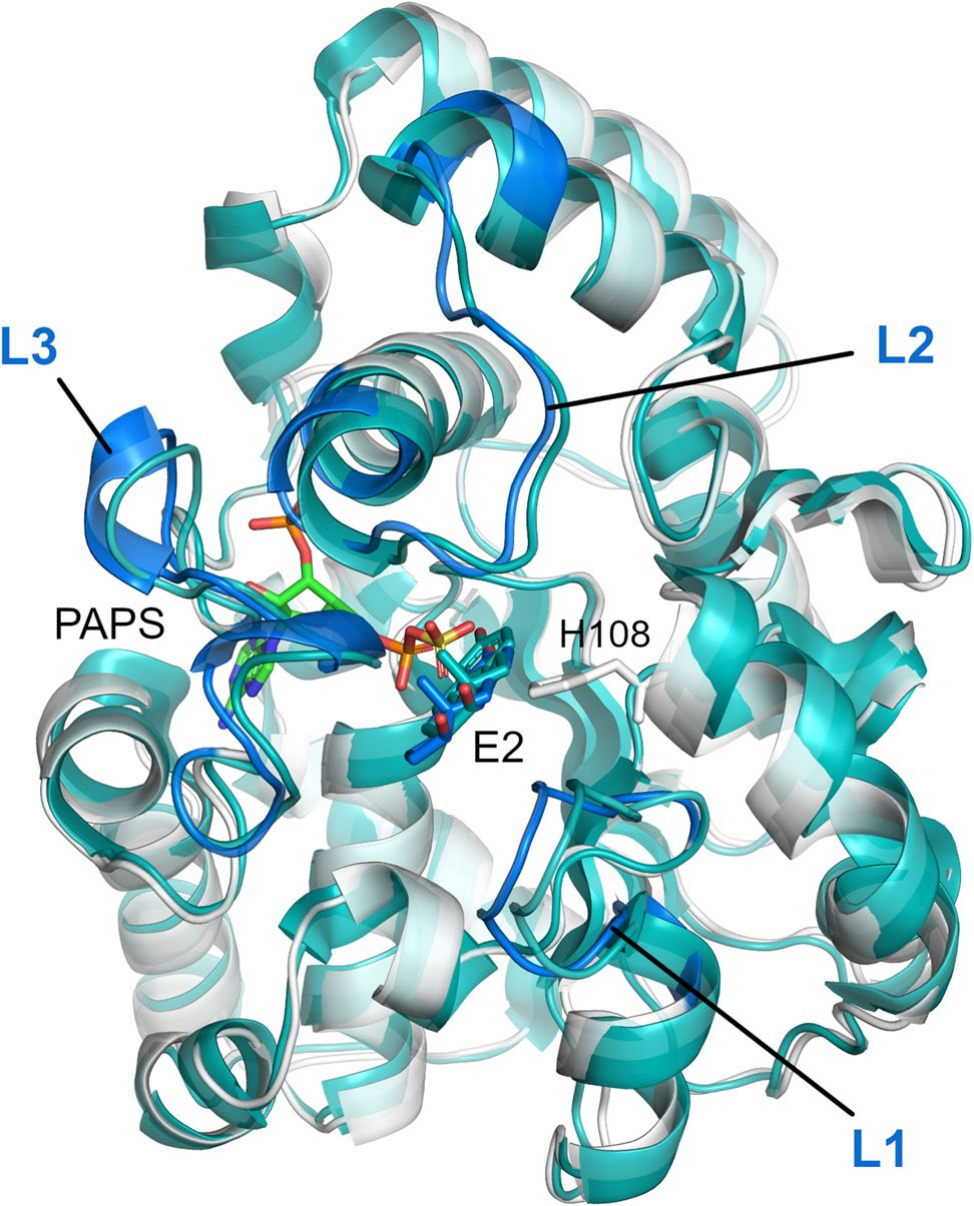
A favorable docking position of E2 in an MDeNM generated conformation (in white) superposed to the crystal structure of SULT1A1*2 co-crystalized with E2 (2D06.pdb –in cyan).

**Figure 8:**
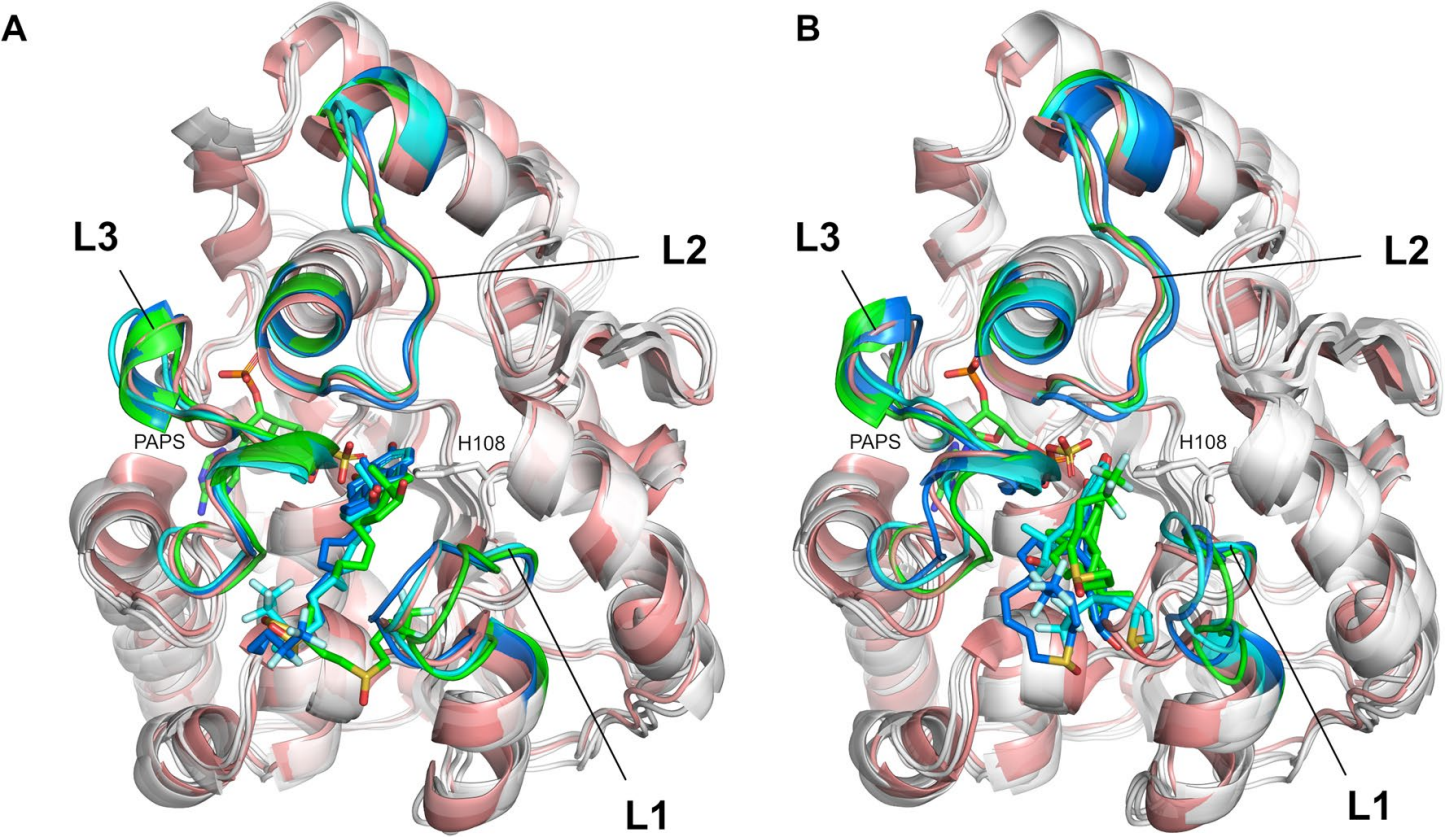
Favorable docking positions of fulvestrant in (**A**) three MD and (**B**) three MDeNM generated conformations. The apo crystal structure of SULT1A1*1 (4GRA.pdb) is shown in salmon for reference.

To further investigate the gating mechanism and substrate recognition of SULT1A1, we additionally analyzed the docking of two estrogens, the substrates 17β-estradiol (E2) and fulvestrant, previously suggested to be accommodated via different mechanisms depending on the co-factor induced isomerization^24^. E2 is a smaller, medium-sized substrate of SULT1A1 that contains a phenolic-hydroxyl group at the C3, and a hydroxyl group at the 17β position. Fulvestrant is an estrogen analogue, a larger substrate of SULT1A1, with an additional 15-atom long functional sidechain at the C7 position. E2 and fulvestrant were both docked into 6000 structures generated by MD and 6000 other structures generated by MDeNM (they were taken every 100 ps during MD and after every second relaxation phase in MDeNM, respectively). The docking poses of both E2 and fulvestrant were considered acceptable on a given enzyme conformation if the BE was lower than − 5 kcal/mol (more favorable binding energy) and the distance between the PAPS sulfate and the ligand’s nucleophilic hydroxyl oxygen was less than 5 Å. Although it has been shown that the formation of fulvestrant-3-sulfate/estradiol-3-sulfate is preferable, it is also possible that low levels of fulvestrant-17-sulfate/estradiol-17-sulfate are produced^43^. The distribution of conformations capable of accommodating E2 and fulvestrant, along the formerly defined distances d(L1,L2) and d(L1,L3), is shown in Fig. 5. MD and MDeNM conformations were capable of accommodating E2, regardless of their openness (Fig. 5B and E), which agrees with previous kinetic and binding studies showing that E2 can bind to open and closed conformations of SULT1A1^23^. The analysis of the conformations showing the strongest BEs (having a BE to estradiol lower than − 10 kcal/mol; denoted by blue ‘x’) further indicates that the extremely closed state is mostly unfavorable even for estradiol binding. This is in line with the fact that E2 is a medium-size substrate of SULT1A1. Fulvestrant showed, even more, an obvious preference towards open conformations. Similarly to MD, as mentioned above, the opening along d(L1,L2) and d(L1,L3) is restricted by the high correlation between them; hence opening along both distances is required for fulvestrant to dock (Fig. 5C). MDeNM results reveal, however, that the opening along d(L1,L3) rather than d(L1,L2) is essential for fulvestrant (Fig. 5F). Analysis of the best docking results of fulvestrant (having a BE lower than − 10 kcal/mol; denoted by blue ‘x’) further confirmed that only conformations with a great d(L1,L3) distance are favorable for fulvestrant docking. MDeNM simulations were capable of generating widely open conformations accessible for fulvestrant, 3 Å along d(L1,L3) beyond MD conformations. Both MD and MDeNM results confirm that, open conformations are still available for big ligands to bind even with the co-factor bound. The distribution of conformations shown in Fig. 5 were also transformed in Free Energy Landscapes (FEL) according to Eq. 1 (see “Materials and methods”) and are shown in Fig. 6. Interestingly, most of the conformations capable of accommodating competent E2 and fulvestrant are of low free energies. An example of a favorable position of E2 docked into an MDeNM generated conformation (Fig. 7) illustrates the excellent superposition to the bioactive conformation of E2 in the structure of SULT1A1*2 co-crystallized with E2. Figure 8 shows competent docking positions of fulvestrant in three MD and three MDeNM generated conformations. Their comparison with the crystal structure of apo SULT1A1*1 (PDB ID 4GRA) demonstrates the utility of using MDeNM simulations, suggesting a larger opening of the pore than observed by the MD simulations and facilitating thus the accommodation of large substrates as fulvestrant.

Further MD simulations were performed for SULT1A1/PAPS bound to a substrate. The best-docked structures for the two substrates E2 and fulvestrant, having the best docking scores and competent positions, were chosen as starting structures for the additional MD simulations. Two docked positions of E2 were chosen, one in an MD—and one in an MDeNM—generated conformations (shown in Fig. 7). For the fulvestrant, three and three starting positions were chosen out of the MD- and MDeNM—generated conformations, respectively (shown in Fig. 8). In 7 out of the 8 MD simulations, the substrate remained in a stable position keeping a distance between the hydroxyl group of the ligand and the sulfate group of PAPS within 5 Å. The unstable fulvestrant-bound complex, starting from an MDeNM conformation, had a significantly different initial substrate orientation compared to the co-crystallized structure of E2 (see in SI Fig. S4F model 2). The binding energies of the two substrates and SULT1A1/PAPS calculated with Autodock Vina scoring function for the complexes’ structures before, and after the 100 ns MD simulations are shown in SI Table S2. It is seen that after all MD simulations with a bound substrate, the predicted binding energies for E2 and fulvestrant (SI Table S2) are closer to the experimental ones (SI Table S1) as compared to the energies calculated after docking only (SI Table S2).

To compare the MD simulations with and without bound substrates, the FELs were calculated with respect to the distances d(L1,L2) and d(L1,L3) (see Fig. 6 and SI Fig S4). The energetically most stable states of the MD simulations with a bound substrate correspond in all cases to conformations that are more open than the crystal structure 4GRA.pdb, both for E2 and fulvestrant. Interestingly, both MD and, to a greater extent, MDeNM were able to generate open conformations starting from the apo-state (without a bound ligand) (Fig. 6), corresponding to these energetically stable MD states in the presence of a bound substrate. Except for the one unstable MD simulation in the presence of fulvestrant as discussed above, both MD simulations with estradiol, and the other five MD simulations with fulvestrant show the induced further opening of the loops in the presence of a bound substrate.

These results are in agreement with previous indications that SULT undergoes a large opening to accommodate very large SULT substrates such as fulvestrant, 4-hydroxytamoxifen, or raloxifene^24,44,45^. However, we should note that the above discussed open SULT1A1/PAPS structures were generated in the presence of PAPS in our case. Thus, our simulations do not entirely support the assumption that recognition of large substrates is dependent on a co-factor isomerization as proposed in^24,25^. Furthermore, allosteric binding was previously proposed to occur for some inhibitors in one part of the large cavity, assuring the substrates' access close to the co-factor^46^. Previous studies suggested that inhibitors like catechins (naturally occurring flavonols)^46^ or epigallocatechin gallate (EGCG)^22^ might inhibit SULT1A1 allosterically close to that cavity. Detailed analysis of our MDeNM results on the flexibility of this large cavity area – constituted by the active site and the pore (also called the catechin-binding site^21^), sometimes accommodating a second inhibitor molecule (e.g. p-Nitrophenol, see PDB ID 1LS6^37^) – showed that some L1 and L3 conformations (e.g. seen in Fig. 8B) ensure sufficient opening of the pore to accommodate large inhibitors like EGCG, and thus such binding into the pore^21,22^ might not be considered as allosteric.

## Conclusion

In this study, we employed MD simulations and the recently developed MDeNM approach to elucidate the molecular mechanisms guiding the recognition of diverse substrates and inhibitors by SULT1A1. MDeNM allowed exploring an extended conformational space of PAPS-bound SULT1A1, which has not been achieved by using classical MD. Our simulations and analyses on the binding of the substrates estradiol and fulvestrant demonstrated that large conformational changes of the PAPS-bound SULT1A1 could occur independently of the co-factor movements. We argue that the flexibility of SULT1A1 ensured by loops L1, L2, and L3 in the presence of the co-factor is extremely high and may be sufficient for significant structural displacements for large ligands, substrates, or inhibitors. Such mechanisms can ensure the substrate recognition and the SULT specificity for various ligands larger than expected, as exemplified here with fulvestrant. Altogether, our observations shed new light on the complex mechanisms of substrate specificity and inhibition of SULT, which play a key role in the xenobiotics and Phase II drug metabolism^2,8^. In this direction, the results obtained using the MDeNM simulations were valuable and highlighted the utility of including MDeNM in protein–ligand interactions studies where major rearrangements are expected.

## Materials and methods

### Protein structures preparation

Some studies indicate that the SULTs are half-site reactive enzymes, and when the nucleotide is bound at only one subunit of the SULT dimer, the “Cap” of that subunit will spend most of its time in the “closed” conformation^27^. Although the dimer interface is adjacent both to the PAPS binding domain and the active site “Cap” of the SULTs in some X-ray structures (e.g. PDB ID 2D06 , SULT1A1 co-crystallized with PAP and E2), suggesting that the interaction between the two subunits may play a role in the enzyme activity, SULT monomers retain their activity in vitro^22^. Furthermore, in other X-ray structures, a different dimer binding site is observed (e.g. PDB ID 2Z5F, SULT1B1 co-crystallized with PAP). Previously, identical behaviors were observed when simulations were performed with monomers or dimers constructed using the canonical interface^24^. Here, all simulations were performed using monomer structures.

Several crystal structures of SULT1A1 are available in the Protein Data Bank (http://www.rcsb.org). The only available structure of SULT1A1*1 containing R213 and M223 without bound ligand was selected, PDB ID: 4GRA^24^. The co-factor PAP present in the 4GRA structure was replaced by PAPS. The PAPS structure was taken of SULT1E1 (PDB ID: 1HY3^47^) and superposed to PAP in 4GRA.pdb by overlapping their common heavy atoms; the differing sulfate group of PAPS did not cause any steric clashes with the protein. The pKa values of the protein titratable groups were calculated with PROPKA^48^, and the protonation states were assigned at pH 7.0. PAPS parameters were determined by using the CHARMM General Force Field 2.2.0 (CGenFF)^49^. The partial charges of PAPS were optimized using quantum molecular geometry optimization simulation (QM Gaussian optimization, ESP charge routine^50^) with the b3lyp DFT exchange correlation functional using the 6–311 + g(d,p) basis set.

A rectangular box of TIP3 water molecules with 14 Å in all directions from the protein surface (82 Å × 82 Å × 82 Å) was generated with CHARMM-GUI^51,52^, and the NaCl concentration was set to 0.15 M, randomly placing the ions in the unit cell. The solvated system was energy minimized with progressively decreasing harmonic restraints applied to atomic positions: steepest descent (SD) was first used where the harmonic force constant was decreased every 100 steps adopting the values 50, 10, 1, and 0.1 kcal/mol/Å^2^. The system was further minimized without harmonic restraints by performing successive cycles of SD and Adopted Basis Newton–Raphson (ABNR) minimizations till a tolerance of RMS energy gradient of 0.01 kcal/mol/Å was reached. The minimization was performed with CHARMM^53^ using the additive all-atom CHARMM force field C36m^54^. The system was then heated and equilibrated at 300 K for 100 ps in an NVT ensemble followed by a 5 ns NPT run at 1 atm pressure. The equilibration was performed with NAMD^55^ using the additive all-atom CHARMM force field C36m^54^. For constant temperature control Langevin dynamics was used with a damping coefficient of 1 ps^−1^. The constant pressure was achieved by using Nose–Hoover method with a piston oscillation period of 50 fs, and a piston oscillation decay time of 25 fs. The integration time step was set to 2 fs. For the energy calculations, the dielectric constant was set to 1. The particle mesh Ewald (PME) method was used to calculate the electrostatic interactions with a grid spacing of 1 Å or less having the order of 6. The real space summation was truncated at 12.0 Å, and the width of Gaussian distribution was set to 0.34 Å^−1^. Van der Waals interactions were reduced to zero by ‘switch’ truncation operating between 10.0 and 12.0 Å.

### MD simulations

MD simulations were carried out with NAMD^55^ using the all-atom CHARMM force field C36m^54^. Three parallel 200 ns long MD simulations were performed for SULT1A1/PAPS without bound ligand starting from the equilibrated structure, with random velocities assigned according to the Maxwell–Boltzmann distribution at 300 K. A time step of 2 fs was used, with the coordinates saved every 10 ps. The parameters for the 200 ns runs were identical to those used for the previously described NPT equilibration of 5 ns. Additional 8 MD simulations of 100 ns were performed for SULT1A1/PAPS in the presence of a bound substrate (E2 and fulvestrant) starting from different substrate positions. The parameters of E2 and fulvestrant were determined by CGenFF. The same MD protocol was then applied as detailed above for SULT1A1/PAPS without bound substrate.

### MDeNM simulations

MDeNM simulations and analyses were performed with CHARMM^53^ using the all-atom CHARMM force field C36m^54^. Starting from the same equilibrated SULT1A1 structure in solution as for the MD simulations, the MDeNM approach was used to map its conformational surface^32^ thoroughly. The equilibrated structure was first energy minimized to calculate the normal modes. For energy minimization, we first used the steepest descent (SD) method with harmonic restraining potentials applied to atomic positions whose force constant were decreased from 10, 1, 0.1, and 0 kcal/mol/Å^2^ every 500 steps. It was followed by the Adopted Basis Newton–Raphson minimization to reach an RMS energy gradient of 10^–5^ kcal/mol/Å. The normal modes of the energy minimized structure were calculated using the VIBRAN module of CHARMM^56^. For the MDeNM calculations, the three low-frequency normal modes contributing the most to the highest RMSF of atomic displacements were taken.

Then, random linear combinations of these modes were generated such that the RMSDs between 1 Å displaced structures along these combined NM directions were greater than 0.3 Å. This provided the directions for unbiased coverage of the large-scale conformational space of the protein. In total, 240 different directions were created. For each of them, MD simulations were performed within which the motion described by the combined NM vector was kinetically promoted; this was achieved by adding to the current MD velocities an additional velocity in the direction of the NM combined vector corresponding to an overall 2 K increase of the system’s temperature. As the excitation energy rapidly dissipates in less than 1 ps, a series of 50 consecutive excitations were achieved after every 4 ps of the MD simulation to allow the system to evolve and relax. Thus, the total MDeNM simulation time was 240 × 50 × 4 ps = 48 ns. The other MD parameters were the same as the given ones in the previous paragraph on *“MD simulations”.*

### Clustering

The Quality Threshold (QT) algorithm^57^ as implemented in VMD^58^ was applied to perform conformational clustering of the MD generated conformations. A distance function defined as the RMSD difference calculated for the heavy atoms of the binding pocket (see in SI for its definition) was used with the maximum cluster diameter set to 1.1 Å. The centers of the 94 most populated clusters containing 85% of all the conformations were then used to dock known substrates and inhibitors of SULT1A1. In the case of the MDeNM generated conformations, the population of clusters is biased due to the common starting structure for each replica and the applied RMSD filtering upon the generation of the excitation directions. A pseudo-uniform selection from all the MDeNM generated conformations was applied with a spacing of 1.1 Å in the RMSD space defined by residues within the binding pocket to create a representative set. A total of 86 structures were retrieved and used for the docking of known substrates and inhibitors of SULT1A1.

### Docking

Docking experiments were performed with AutoDock Vina 1.1.2^59^ that employs gradient-based conformational docking and an empirical scoring function predicting the protein–ligand binding energy in kcal/mol. A list of 132 known substrates and inhibitors of SULT1A1 were taken, collected in our previous work^10^ and^28,41^. The protein conformations selected for docking were pre-processed with AutoDockTools^60^, the solvent was removed, non-polar hydrogens were merged, and Gasteiger charges were assigned. The ligands were prepared for the docking using AutoDockTools. A grid box of 24 Å × 24 Å × 24 Å was centered on the binding pocket with a spacing of 1 Å. The grid center was set to x = 27.050 Å; y = 17.520 Å; z = 17.653 Å with respect to the crystal structure 4GRA.pdb. The maximum number of binding modes was set to 20, the exhaustiveness of the global search to 10, the maximum energy difference between the retained best and worst binding modes to 15 kcal/mol. During the docking, the ligands and the binding site residues K106 and F247 observed to change their side-chain conformations easily during the MD and MDeNM simulations were handled flexibly; the rest of the protein and the co-factor were kept rigid.

### Free Energy Landscape (FEL) analysis

FELs of conformations corresponding to the different MD and MDeNM simulations were calculated within the plane defined by the distances d(L1,L2) and d(L1,L3). The most populated state was used as a reference for calculating free energy differences. The free energy difference (ΔG_α_) of a given state *α* was determined by considering the probability of the occurrence of the two states *P*(*q*_*α*_) and *P*_*max*_(*q*) given by the equation:

1 $$ \Delta G_{\alpha }  =  - k_{B} T\ln \left[ {\frac{{P\left( {q_{\alpha } } \right)}}{{P_{{\max }} \left( q \right)}}} \right] $$

where *k*_*B*_ is the Boltzmann constant, *T* is the temperature of the simulation, *P(q*_*α*_*) is* an estimate of the probability density function obtained from the bi-dimensional histogram of the conformations distribution in the plane of d(L1,L2) and d(L1,L3) during the simulation. *P*_*max*_*(q)* is the probability of the most populated state.

## Supplementary Information

Supplementary Information.

## Data Availability

All data generated during this study are included in this published article and its Supplementary Information file.
