# Supplementary material for: Insights into the substrate binding mechanism of SULT1A1 through molecular dynamics with excited normal modes simulations
Source: Sci Rep. 2021 Jun 23;11:13129. doi: 10.1038/s41598-021-92480-w (PMC8222352; doi:10.1038/s41598-021-92480-w)
Supplement: Supplementary file 1 — Supplementary Information. [file 41598_2021_92480_MOESM1_ESM.pdf]

Supporting Information

Insights into the substrate binding mechanism of SULT1A1 through Molecular Dynamics with excited Normal Modes simulations

B. Dudas[1,2,#], D. Toth[3,#], D. Perahia[2], A. B. Nicot[4], E. Balog[3,*], M. A. Miteva[1,*]

[1]Inserm U1268 MCTR, CiTCoM UMR 8038 CNRS - University of Paris, Paris, France
[2]Laboratoire de Biologie et Pharmacologie Appliquée, Ecole Normale Supérieure Paris-Saclay, UMR 8113, CNRS, Gif-sur-Yvette, France
[3]Department of Biophysics and Radiation Biology, Semmelweis University, Budapest, Hungary
[4]Inserm, Université de Nantes, Centre de Recherche en Transplantation et Immunologie, UMR 1064, ITUN, F-44000 Nantes, France

[#]1st coauthors
[*]corresponding authors: maria.mitev@inserm.fr, balog.erika@med.semmelweis-univ.hu

SI Figures

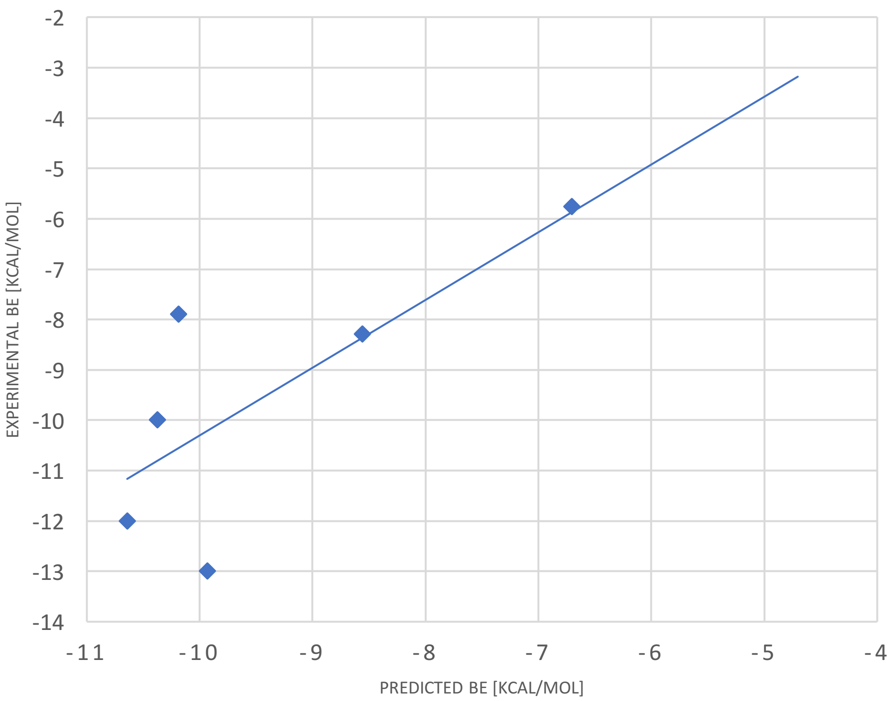

Figure S1. Predicted and experimental binding energy correlation for SULT1A1 ligands. Predicted binding energies (BE) were calculated by averaging over the best scored Autodock Vina energies obtained for the best 10 MD conformations and the best 10 MDeNM conformations. The experimental binding energies (BE) were taken or calculated using ligand affinity constants as reported in the literature (see SI Table S1).

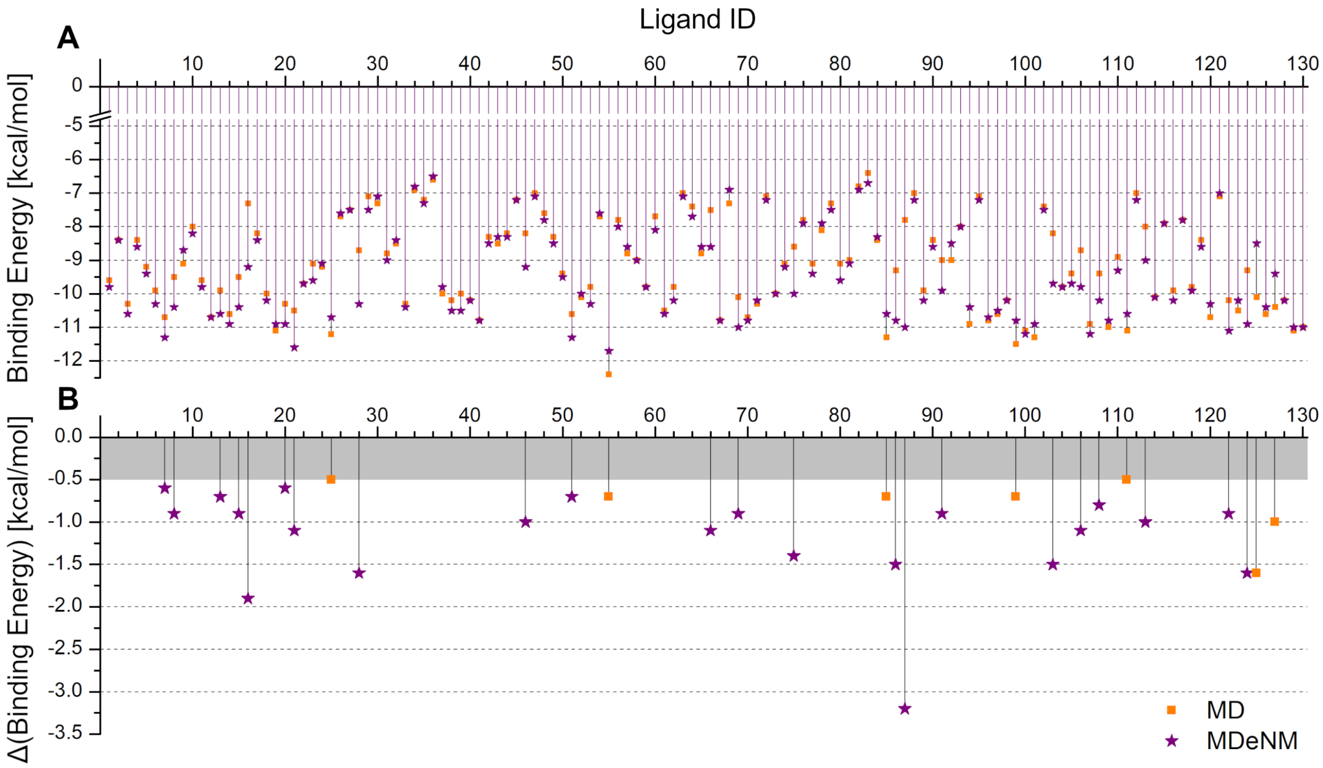

Figure S2. A. The best retained BE for each of the 132 known ligands over the MD (denoted by orange squares) and MDeNM (denoted by purple stars) conformational ensemble and B. the differences between the best BEs retained by MD and MDeNM conformations with the 5 Å distance criterion applied to the substrates. For the better visualization, only differences larger than 0.5 kcal/mol are indicated.

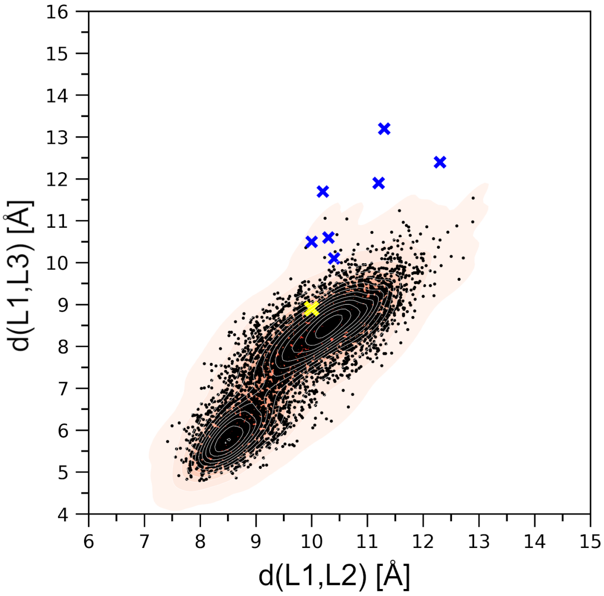

Figure S3. Distribution in the plane defined by d(L1,L2) vs. d(L1,L3) distances of all the MD generated structures (black dots) and the MDeNM structures (blue 'x'-es) that can accommodate competent orientations of bigger ligands with BEs inaccessible for any MD generated conformation. The location of the crystal structure (4GRA.pdb) is shown in yellow 'x'.

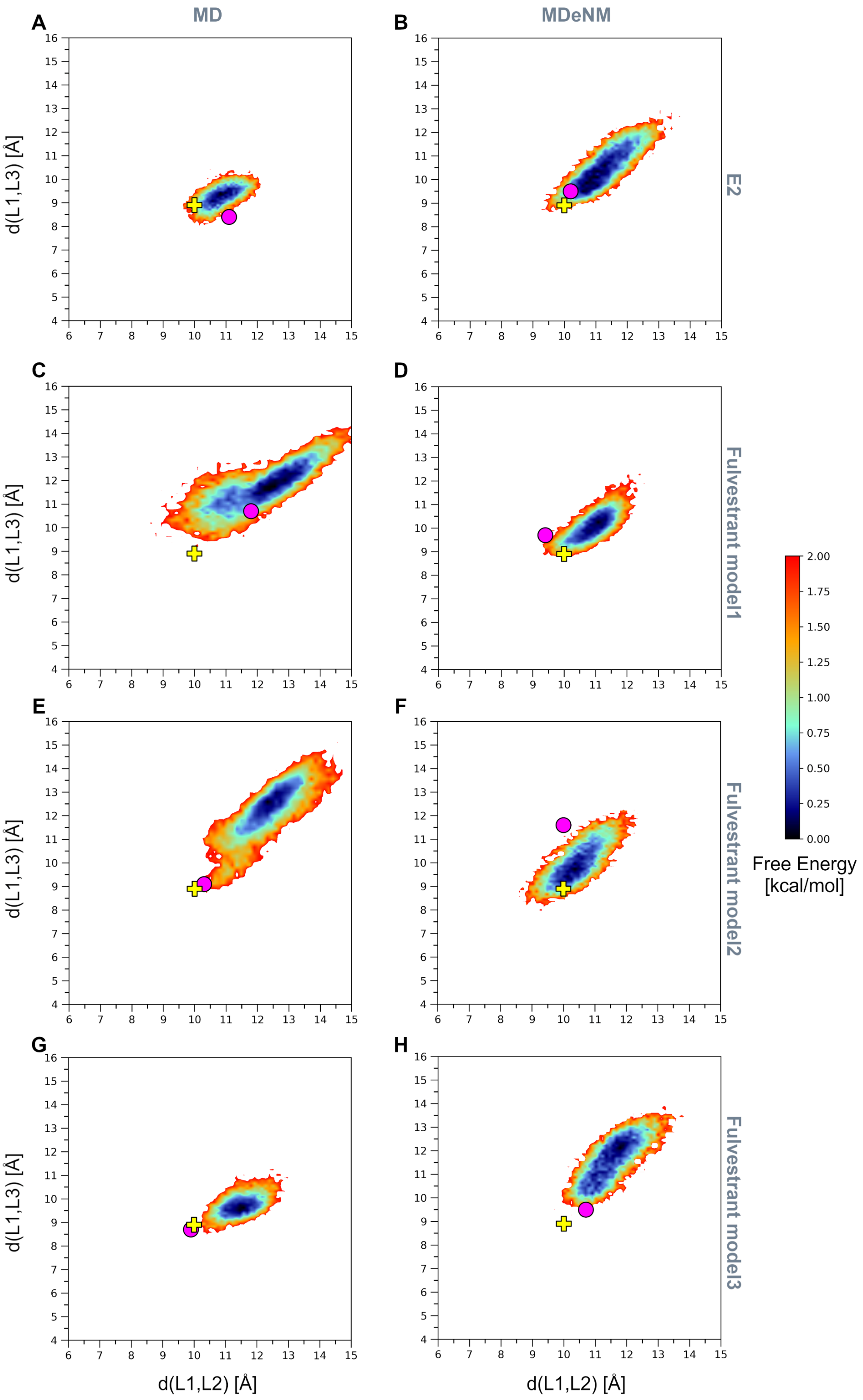

Figure S4. Free Energy Landscapes (FELs) of the complexes substrate-SULT1A1/PAPS in the space defined by the distances d(L1,L2) and d(L1,L3) of the 100 ns long MD simulations starting from an estradiol-bound MD (A) and MDeNM (B) conformations; and from fulvestrant-bound MD (C, E, G) and MDeNM (D, F, H) conformations. All starting complexes were taken after the docking with AutoDock Vina. The crystal structure (4GRA.pdb) is denoted by yellow ‘**+**’. The starting conformation for the MD simulations is denoted by a violet circle.

Table S1. Predicted and experimental binding energies for SULT1A1 ligands. Predicted binding energies were calculated by averaging over the best scored Autodock Vina energies obtained for the best 10 MD conformations and the best 10 MDeNM conformations. The experimental binding energies were taken or calculated using ligand affinity constants as reported in the literature.

Compound	Predicted Binding Energy (kcal/mol)	Experimental Binding Energy (kcal/mol)	REF
apomorphine	-9.9	-13.0	1
ethinyl estradiol	-10.6	-12.0	2
p-nitrophenol	-6.7	-5.8	3
4-hydroxytamoxifen	-8.6	-8.3	4
17β-estradiol (E2)	-10.4	-10.0	1
fulvestrant	-10.2	-7.9	5

1. Thomas, N. L., and Coughtrie, M. W. (2003) Sulfation of apomorphine by human sulfotransferases. Evidence of a major role for the polymorphic phenol sulfotransferase, SULT1A1. Xenobiotica 33, 1139–1148
2. Rohn, K. J., Cook, I. T., Leyh, T. S., Kadlubar, S. A., and Falany, C. N. (2012) Potent inhibition of human sulfotransferase 1A1 by 17 -ethinylestradiol. Role of 3 -phosphoadenosine 5 -phosphosulfate binding and structural rearrangements in regulating inhibition and activity. Drug. Metab. Dispos. 40, 1588–1595
3. Lu-Yi Lu, Han-Ping Chiang, Wei-Ti Chen, and Yuh-Shyong Yang Dimerization Is Responsible for the Structural Stability of Human Sulfotransferase 1A1. DRUG METABOLISM AND DISPOSITION. 37:1083–1088, 2009
4. Ting Wang, Ian Cook, and Thomas S. Leyh , 3′-Phosphoadenosine 5′-Phosphosulfate Allosterically Regulates Sulfotransferase Turnover, Biochemistry 2014, 53, 6893–6900
5. Cook, I., Wang, T., Almo, S. C., Kim, J., Falany, C. N., and Leyh, T. S. (2013) The gate that governs sulfotransferase selectivity. Biochemistry 52, 415–424

Table S2. Binding energies (BE) of SULT1A1 substrates calculated with Autodock Vina scoring function before and after MD simulations of 100 ns starting from 8 different substrate-SULT1A1/PAPS structures obtained by docking.

Substrate	Starting SULT1A1/PAPS Conformation taken from	Complex No.	BE (before MD) [kcal/mol]	BE (after MD) [kcal/mol]
E2	MD	1	-11.0	-10.8
	MDeNM	1	-11.4	-10.4
Fulvestrant	MD	1	-10.7	-8.8
		2	-10.6	-9.0
		3	-9.9	-8.5
	MDeNM	1	-11.1	-8.1
		2	-10.1	-7.6
		3	-10.0	-9.4

List of residues forming the binding pocket:
I21, F24, T45, Y46, P47, F81, F84, K85, A86, I89, K106, T107, H108, F142, A146, K147, V148, H149, Y169, Y240, T241, T242, V243, P244, Q245, E246, F247, M248, D249, H250, F255
